# Proteomics Analysis of Polyphyllin D-Treated Triple-Negative Breast Cancer Cells Reveal the Anticancer Mechanisms of Polyphyllin D

**DOI:** 10.1007/s12010-023-04679-4

**Published:** 2023-08-25

**Authors:** Chuanchao Wei, Anwei Mao, Yongzhi Liu, Qing Zhang, Gaofeng Pan, Weiyan Liu, Jiazhe Liu

**Affiliations:** 1 Institute of Fudan-Minhang Academic Health System, Minhang Hospital, Fudan University, Shanghai, 201100 China; 2 Department of General Surgery, Affiliated Xiaoshan Hospital, Hangzhou Normal University, Zhejiang, China

**Keywords:** Liquid chromatography-tandem mass spectrometry (LC–MS/MS), Polyphyllin D (PD), Triple-negative breast cancer (TNBC), Oxidative phosphorylation pathway, Spliceosome, Nodal modulator 2/3 (NOMO2/3)

## Abstract

Polyphyllin D (PD), one of the important steroid saponins in traditional medicinal herb *Paris polyphylla*, has been demonstrated to have anticancer activity both in vitro and in vivo. However, the mechanisms through which PD exerts its anticancer effects in triple-negative breast cancer (TNBC) remain unclear. Our study was presented to evaluate the anticancer effect and the potential mechanisms of PD in two TNBC cell lines, BT-549 and MDA-MB-231. Through comprehensively comparing the liquid chromatography-tandem mass spectrometry (LC–MS/MS) data of PD-treated and untreated BT-549 and MDA-MB-231 cells, we found that PD could induce apoptosis of TNBC cells by activating oxidative phosphorylation pathway in BT-549 cells, as well as inhibiting spliceosome function alteration in MDA-MB-231 cells. These results suggested that the mechanisms underlying the pro-apoptotic effect of PD on TNBC may be cell type-specificity-dependent. Moreover, we found that nodal modulator 2/3 (NOMO2/3) were downregulated both in PD-treated BT-549 and MDA-MB-231 cells, suggesting that NOMO2/3 may be the potential target of PD. Verification experiments revealed that PD deceased NOMO2/3 expression at protein level, rather than mRNA level. Whether NOMO2/3 are the upstream modulators of oxidative phosphorylation pathway and spliceosome needs further validation. In conclusion, a comprehensive proteomics study was performed on PD-treated or untreated TNBC cells, revealing the anticancer mechanisms of PD.

**Supplementary Information:**

The online version contains supplementary material available at 10.1007/s12010-023-04679-4.

## Introduction

As one of the most common female malignancy, breast cancer (BC) is the major cause of death in women [1]. According to the expression of estrogen receptor (ER), progesterone receptor (PR), and human epidermal growth factor receptor type 2 (HER2), BC is divided into at least four subtypes: Luminal A (ER + /PR + / HER2 −), Luminal B (ER + /PR + /HER2 +), HER2 (ER − /PR − /HER2 +) and triple-negative breast cancers (TNBC, ER − /PR − /HER2 −) [2]. TNBC is the subtype with higher aggressivity and worse prognosis compared to other subtypes, accounting for 10 − 15% of all breast cancer cases [3]. Besides surgery and radiotherapy, chemotherapy, endocrine therapy and targeted therapy are conventional treatment for BC. Nevertheless, they are inappropriate for TNBC, because of the lack of specific receptors [4]. To solve this problem, novel drugs that target TNBC, specifically the receptor-independent drugs, are being widely investigated [5, 6].

Polyphyllin D (PD) is a kind of steroid saponin, which is extracted from a traditional Chinese medicine *Paris polyphylla* classified as a member of lily family [7]. Its anticancer effects have been reported in liver, bladder, pancreatic, breast, prostate and neuroblastoma [7–13]. However, the underlying mechanism is complicated. For example, PD is demonstrated to induce cell death in neuroblastoma cell lines in a cell type-specific manner. To be specific, cell death in NB-69 cells was caused by apoptosis, whereas PD-induced IMR-32 and LA–N-2 cell death mainly through necroptosis [7]. In our previous study of PD against BC, we find that PD induces BC cell death through JNK1/Bcl-2 pathway-mediated apoptosis [14]. However, some preliminary results indicate that there are still other pathways uncovered, prompting us to comprehensively analyze the underlying mechanisms.

Proteomic profiling is a promising approach to identify the alterations of proteins at whole-cell level, which is widely used in the study of cancer, drug development and data mining [15]. Siu et al. performed 2-DE-based proteomic study of PD on human non-small cell lung cancer (NSCLC) for the first time, and identified PD as a potential ER stress inducer [16]. However, due to the limitations of technology, only 14 differential expression proteins (DEPs) were identified. Herein, liquid chromatography-tandem mass spectrometry (LC–MS/MS) was performed on PD-treated or untreated BT-549 and MDA-MB-231 cells, and 7123 proteins were probed in total, with 6958 and 6673 proteins being identified in BT-549 groups and MDA-MB-231 groups, respectively. The proteome results with such a depth coverage provide us an opportunity to study the role of PD at the whole genome-wide level. We found that PD could induce apoptosis of TNBC cells by activating oxidative phosphorylation pathway in BT-549 cells, as well as inhibiting spliceosome function alteration in MDA-MB-231 cells. These results suggested that the mechanisms underlying the pro-apoptotic effect of PD on TNBC may be cell type–specificity-dependent. We also identified nodal modulator 2/3 (NOMO2/3) as the targets of PD, and certified that PD deceased the expression of NOMO2/3 at protein level in verification experiments.

## Materials and Methods

### Cell Culture

Human TNBC cell lines, BT-549 and MDA-MB-231, were purchased from the American Tissue Culture Collection (ATCC, Manassas, VA, USA). BT-549 cells or MDA-MB-231 cells were maintained in RPMI-1640 medium (Corning, NY, USA) or Dulbecco’s Modified Eagle Medium (DMEM; Gibco, Grand Island, NY, USA) with 10% (v/v) fetal bovine serum (FBS; Hyclone US origin, GE Lifescience) and 1% penicillin/streptomycin (Gibco-BRL), respectively. These two cell lines were maintained in a humidified atmosphere with 5% CO_2_ at 37 °C.

### Cell Proliferation and Cell Viability Assay

CellTiter-Glo Luminescent Cell Viability assay (Promega Corporation, Madison, WI, USA) was introduced to examine cell proliferation according to the manufacturer’s instructions. Briefly, cells were seeded in 96-well plates at 1.0 × 10^3^ per well and cultured at 37 °C overnight. Then, 100 µL CellTiter-Glo solution was added into the culture medium and incubated at room temperature for 20 min. Finally, the luminescence intensity was recorded. The 50% inhibitory concentration (IC_50_) was assessed as a percentage of absorbency relative to control cells that treated with DMSO by GraphPad Prism software (San Diego, CA, USA).

### Cell Apoptosis Assay

After being seeded in 24-well cell plates, cells were treated with PD at the indicated concentrations and times. Propidium iodide (PI) (Sigma-Aldrich, P4170) was added at a final concentration of 50 μg/mL to measure the dead cells with damaged cell membrane integrity.

### Liquid Chromatography-Tandem Mass Spectrometry (LC–MS/MS)

For each group, total protein was extracted and digested using filter-acid sample preparation (FASP) method. Then, proteome analysis was processed on a nanoElute-HPLC System (Bruker Daltonics) coupled with a hybrid trapped ion mobility spectrometry quadrupole times-of-flight mass spectrometer (TIMS-TOF Pro Bruker Daltonics, Billerica, MA, USA) via a Captive Spray nano-electrospray ion source. MS raw files were searched against the Swiss-Prot database (downloaded on August 20, 2020, containing 20,375 protein sequence entries) using PEAKS Online Xpro Software (v1.4) for peptide and protein identifications. The detail information is presented in the Supplementary information. The mass spectrometry proteomics data have been deposited to the ProteomeXchange Consortium (http://proteomecentral.proteomexchange.org) via the iProX partner repository [17, 18] with the dataset identifier PXD040990.

### Differential Expression Protein Identification and Pathway Enrichment Analysis

The proteins with the threshold of fold change ≥ 2 (or ≤ 0.5) and *p* value < 0.05 (two-sided Student’s *t* test) were identified as differential expression proteins (DEPs) between the treatment group and control group. Gene set enrichment analysis (GSEA) was used for pathway enrichment analysis using KEGG gene sets (C2) [19]. FDR < 0.05 was set as a cutoff. All the ranked list was used to assess the distribution of the proteins in each gene set across the proteome data.

### Clinical Proteomic Tumor Analysis Consortium (CPTAC) Databases

The comparison of NOMO expression between BC subtypes and non-cancerous normal tissues was determined using the CPTAC database (https://cptac-data-portal.georgetown.edu/cptacPublic) and the University of ALabama at Birmingham CANcer data analysis Portal (UALCAN, https://ualcan.path.uab.edu/analysis-prot.html).

### RNA Extraction and Quantitative RT-PCR (qRT-PCR)

Total RNA was extracted from cells using the RNAiso Plus reagent (Takara Biotechnology Co., Ltd, Dalian, China). Then, TransScript First-Strand cDNA Synthesis SuperMix (Transgene Biotech, AT301-02) was used to obtain cDNA. qRT-PCR was conducted using SYBR® Premix Ex Taq Kit (TAKARA) to detect the mRNA expression levels of NOMOs. β-actin was used as the housekeeping gene. The primers for NOMOs and β-actin were purchased from Thermo Fisher (Hs00276262_m1, Catalog #: 4448892; Hs01060665_g1, Catalog #: 4331182).

### Plasmid and Transfection

The NOMO2 expression vector pCMV3-NOMO2 was purchased from SinoBiological (HG24005-UT). Transient transfections were performed by using Lipofectamine 2000 (Invitrogen) according to the manufacturer’s instructions.

### Western Blot

The cells were lysed on ice using RIPA lysis buffer (Applygen, China) supplemented with PMSF and Protease Inhibitor Cocktail (PIC) and then quantified using the BCA Protein Assay Kit. 20 mg of the cell protein lysates were subjected to SDS–polyacrylamide gel electrophoresis (SDS-PAGE). After the conventional methods for protein separation and transfer, the membrane was incubated with primary antibodies (anti-NOMO2, 1:2000, Thermo Fisher, PA5-100644; anti-β-actin, 1:5000, A5316, Sigma) at 4 °C overnight and secondary antibody at room temperature for 1 h. The bands were visualized by Typhoon FLA 9500 (GE Healthcare) image scanning.

### Statistical Analysis

Results were analyzed using GraphPad Prism 8.0. Statistical analyses were performed using bilateral Student’s *t* test (unpaired). All data shown represent the results obtained from triplicated independent experiments with standard errors of the mean (mean ± SD). *P* < 0.05 was considered as statistical significance.

## Results

### PD Inhibits Cell Viability and Induces Apoptosis in TNBC Cells

**Fig. 1:**
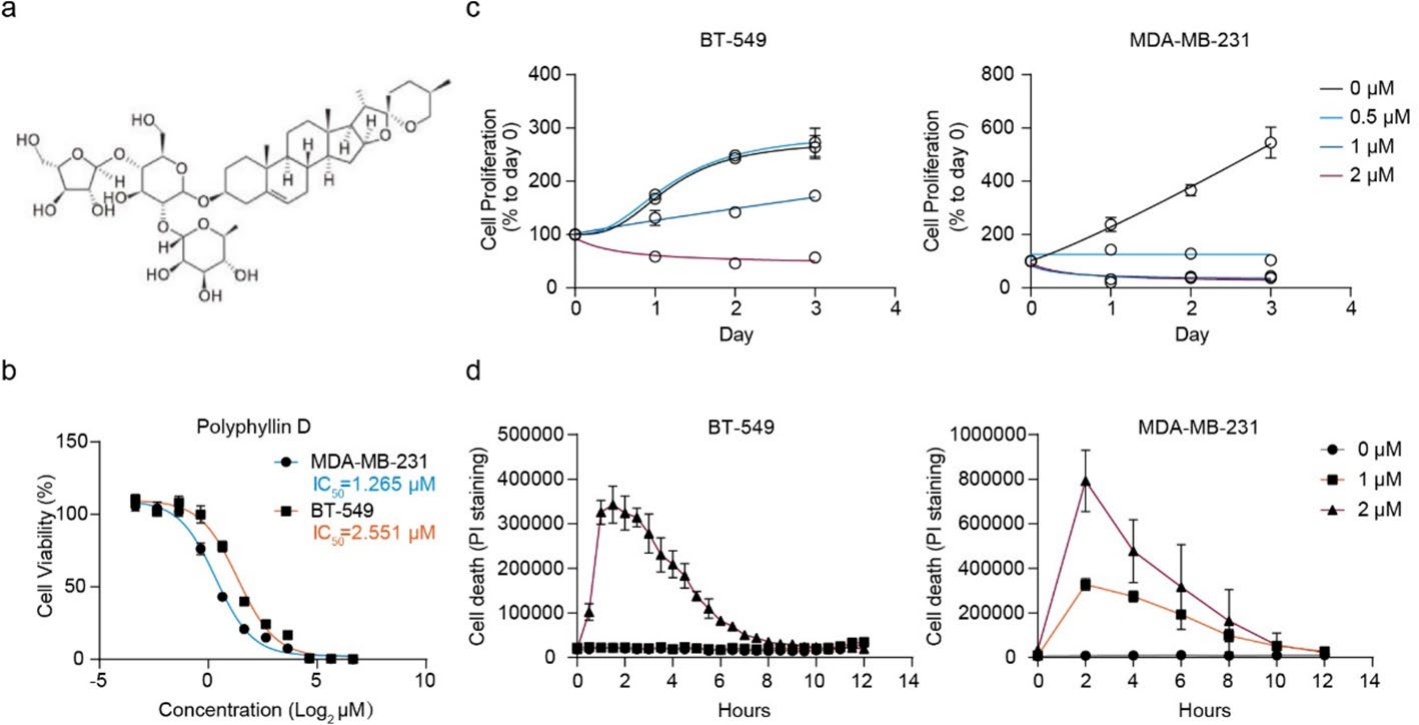
PD inhibits cell viability and induces apoptosis in TNBC cells. **a** Chemical structure of Polyphyllin D. **b** The resulting IC_50_ values upon PD treatment of the individual tested cell lines after 24 h. The IC_50_ of PD were 1.265 μM for MDA-MB-231 cells and 2.551 μM for BT-549 cells, respectively. c BT-549 and MDA-MB-231 cells were treated with indicated concentrations of PD for 24 h, 48 h, 72 h, and 96 h as indicated. Then, cell viability assay was carried out. **d** PI staining was performed to exam the apoptosis of BT-549 and MDA-MB-231 cells at indicated times. All data were measured in three independent experiments

In our previous study, we found that PD could inhibit BC cell viability [14]. In this study, we first evaluated the cytotoxic effect of PD (Fig. 1a) on human TNBC cell lines, BT-549 and MDA-MB-231. The resulting IC_50_ values after a 24-h co-incubation of TNBC cell lines and PD were 1.265 μM for MDA-MB-231 cells and 2.551 μM for BT-549 cells, respectively (Fig. 1b). To explore the role of PD on TNBC cell proliferation, we carried out cell viability assay on BT-549 and MDA-MB-231 cells treated by PD with different concentrations. The results showed that PD could significantly inhibit BT-549 and MDA-MB-231 cell proliferation in dose- and time-dependent manners (Fig. 1c). The PI staining results showed that PD also suppressed TNBC cell proliferation through inducing apoptosis, and could trigger TNBC cells acute death most significantly at 2 h (Fig. 1d). The findings above demonstrated the anticancer effects of PD on TNBC cells.

### The Proteome Atlases of BT-549 and MDA-MB-231 Cells

**Fig. 2:**
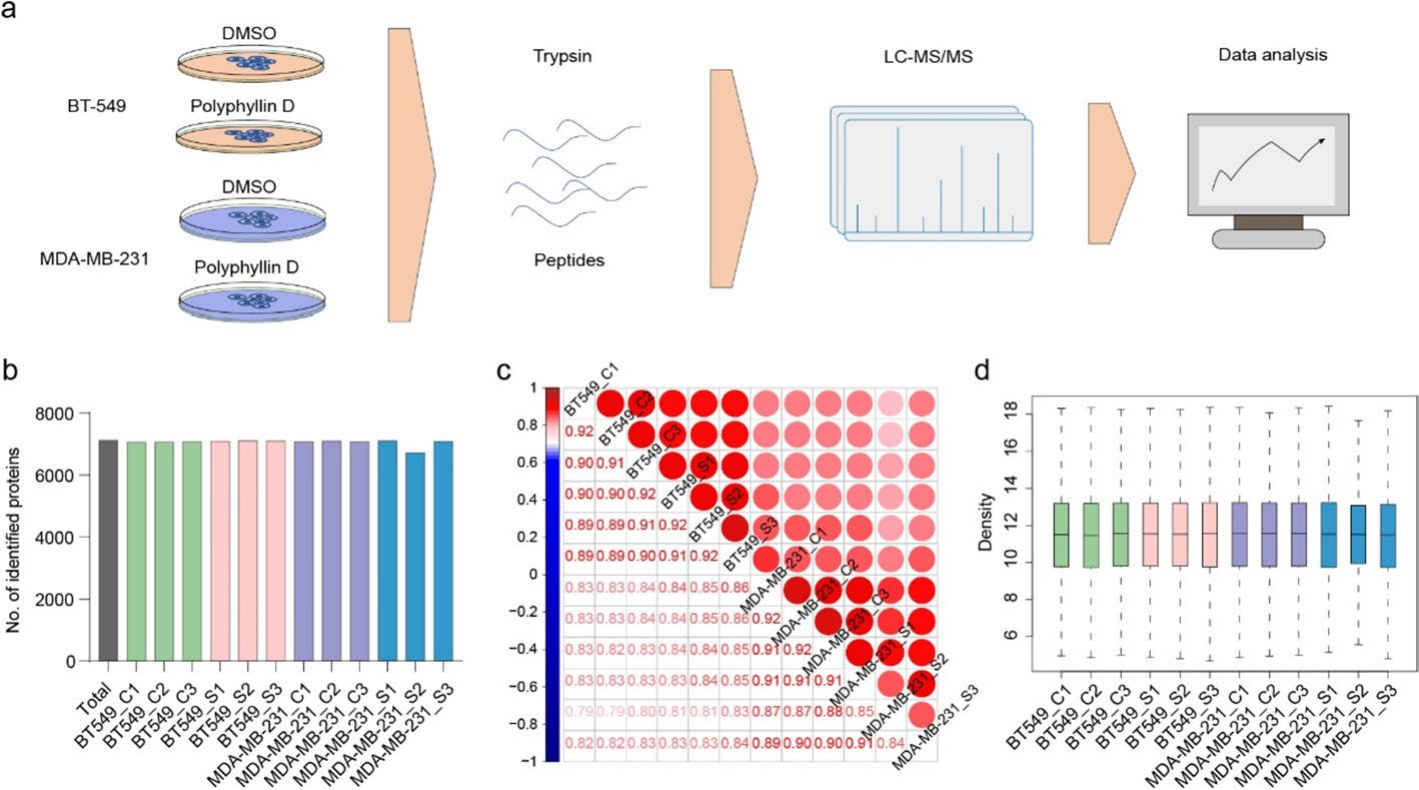
The proteome atlases of BT-549 and MDA-MB-231 cells. **a** Illustrated workflow scheme. **b** Numbers of identified proteins in LC–MS/MS measurements. The proteome analysis probed 7123 proteins in total, with 6958 and 6673 proteins being identified in BT-549 groups and MDA-MB-231 groups, respectively. **c** The average Pearson correlation coefficient *r* of the replicates. The results suggested the high repeatability of our experiments. **d** The quantification results of 12 profiling. The medians and distributions of all profiling were on the same level, indicating the proteome of 12 experiments were parallel comparable

To probe the global spectrum of protein expression properties of PD-treated and untreated TNBC cells, we performed proteome analysis of BT-549 and MDA-MB-231 cells through liquid chromatography-tandem mass spectrometry (LC–MS/MS) after tryptic digestion of cell lysates, followed by protein identification and quantification with the software PEAKS Online (Fig. 2a). The proteome probed 7123 proteins in total, with 6958 and 6673 proteins being identified in BT-549 groups and MDA-MB-231 groups, respectively (Fig. 2b, Table S1). The results of the average Pearson correlation coefficient *r* of the replicates suggested the high repeatability of our experiments (Fig. 2c). The quantification results showed that the medians and distributions of all profiling were on the same level (Fig. 2d), indicating that the proteome of 12 experiments were parallel comparable.

### PD Could Activate Oxidative Phosphorylation Pathway in BT-549 Cells

**Fig. 3:**
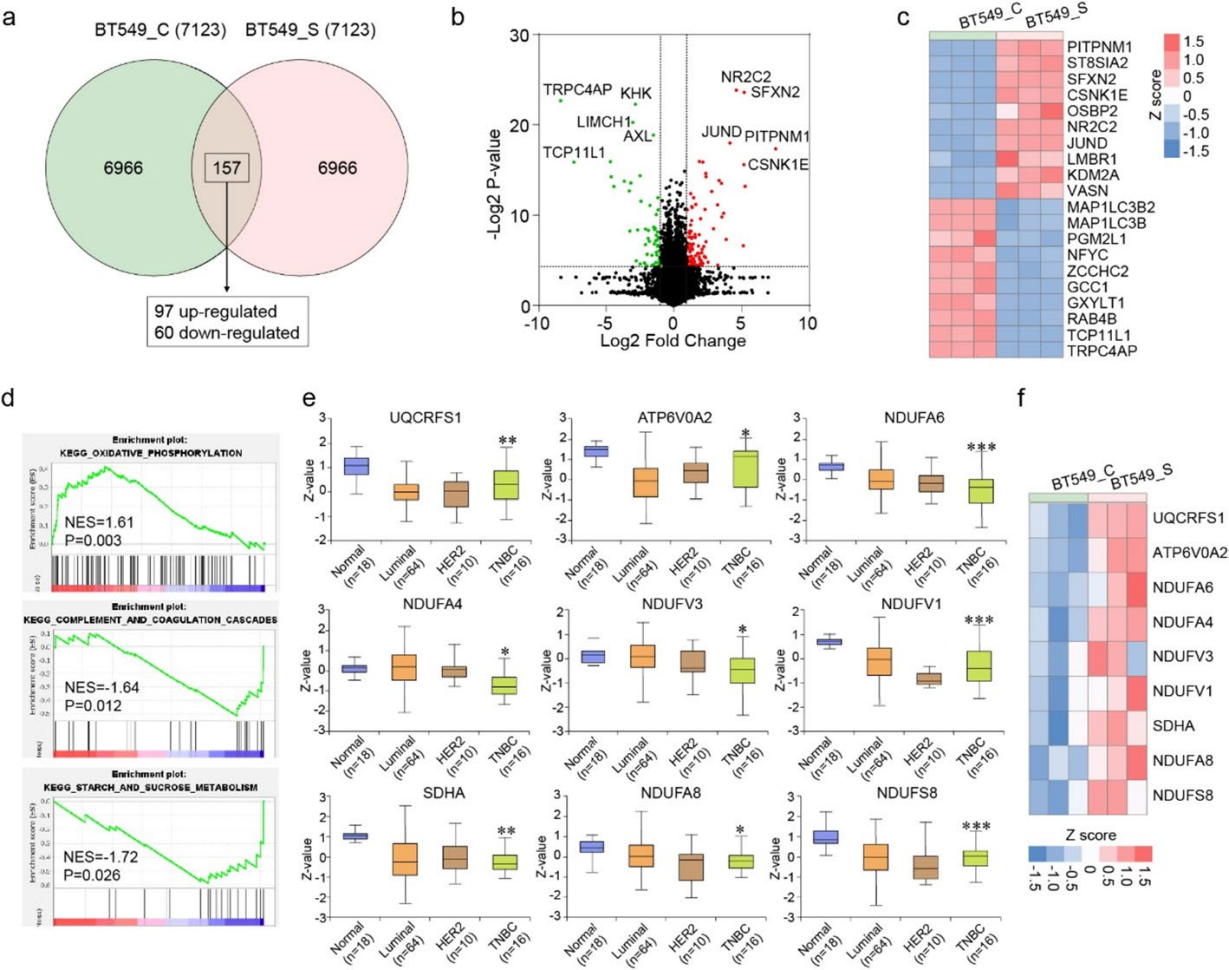
PD could activate oxidative phosphorylation pathway in BT-549 cells. **a** Quantitative Venn diagram showing the proteins with significant alterations. 157 DEPs with the threshold of fold change ≥ 2 (upregulated or downregulated) and *p* value < 0.05, including 97 upregulated proteins and 60 downregulated proteins, were identified. **b** Volcano plots showing proteins with significant differences of proteins upregulated (red dots) or downregulated (green dots). **c** The heat map represents the top 10 significant differences (up- and downregulated) in protein abundance. **d** GSEA revealing pathways identified between PD-treated and untreated BT-549 cells. **e** The protein expression of proteins identified in oxidative phosphorylation pathway (data from CPTAC). *: *p* < 0.05; **: *p* < 0.01; ***: *p* < 0.001 between normal and TNBC groups. **f** The heat map represents the identified proteins in PD-treated and untreated BT-549 cells

We then studied the differential expression proteins (DEPs) between proteome of PD-treated (S group) and untreated (C group) BT-549 cells. 157 DEPs with the threshold of fold change ≥ 2 (upregulated or downregulated) and *p* value < 0.05 were identified, including 97 upregulated proteins and 60 downregulated proteins (Fig. 3a, Table S2). We exhibited the top 10 DEPs (upregulated and downregulated) with volcano plots and heatmap (Fig. 3b, c). GSEA of the global proteome datasets showed that upregulated proteins were enriched in oxidative phosphorylation pathway in PD-treated cells, whereas downregulated proteins in PD-treated cells were mainly involved in pathways including complement and coagulation cascades, starch and sucrose metabolism (Fig. 3d, Table S3).

It has been demonstrated that normal cells in body primarily utilize oxidative phosphorylation for growth and survival [20]. In contrast to that, cancer cells rewire their metabolism in unique ways, such as aerobic glycolysis, also known as the “Warburg effect”, to rapidly proliferate and survive [21]. In this study, except for the discovery of activation of oxidative phosphorylation pathway in PD-treated BT-549 cells (Fig. 3d), it was noticed that the pathway-related proteins, which were downregulated in TNBC patients (Fig. 3e), were upregulated after PD treatment in BT-549 cells (Fig. 3f). The results suggested that PD could inhibit the proliferation of BT-549 cells by activating oxidative phosphorylation pathway.

### PD Could Reduce Aberrant Splicing in MDA-MB-231 Cells

**Fig. 4:**
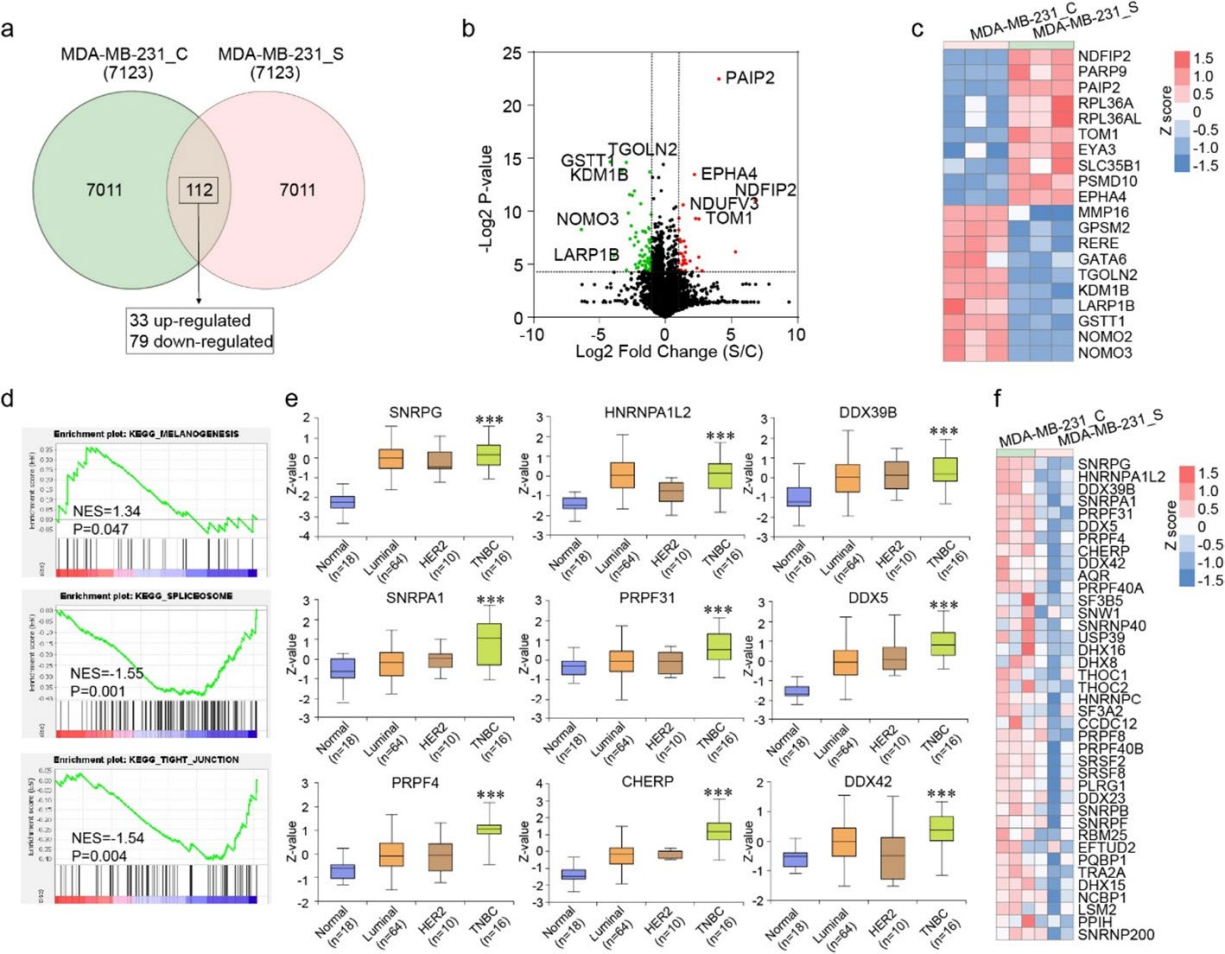
PD could inhibit spliceosome in MDA-MB-231 cells. **a** Quantitative Venn diagram showing the proteins with significant alterations. 112 DEPs with the threshold of fold change ≥ 2 (upregulated or downregulated) and *p* value < 0.05, including 33 upregulated proteins and 79 downregulated proteins, were identified. **b** Volcano plots showing proteins with significant differences of proteins upregulated (red dots) or downregulated (green dots). **c** The heat map represents the top 10 significant differences (up- and downregulated) in protein abundance. **d** GSEA revealing pathways identified between PD-treated and untreated MDA-MB-231 cells. **e** The protein expression of proteins identified in spliceosome function (data from CPTAC). *** *P* < 0.001 between normal and TNBC groups. **f** The heat map represents the identified proteins in PD-treated and untreated MDA-MB-231 cells

Similarly, we next investigated the effect of PD treatment on perturbation of proteome pattern of MDA-MB-231 cells. Table S4 showed the DEPs with the threshold of fold change ≥ 2 (upregulated or downregulated) and *p* value < 0.05 for MDA-MB-231 cells. As shown in Fig. 4a, a total of 112 proteins were observed, including 33 upregulated proteins and 79 downregulated proteins. The top 10 upregulated and 10 downregulated proteins were shown with volcano plots and heatmap in Fig. 4b and c. GSEA demonstrated that proteins upregulated in PD-treated MDA-MB-231 cells were significantly enriched in melanogenesis, whereas proteins downregulated in PD-treated cells were mainly involved in pathways or functions including spliceosome, tight junction, glucose homeostasis (glycolysis and gluconeogenesis), xenobiotics metabolism, N glycan biosynthesis, and histidine metabolism (Fig. 4d, Table S5).

Recent studies have highlighted the frequently altered splicing patterns in cancer, which is mainly caused by dysfunction of proteins in spliceosome pathway [22, 23]. Correspondingly, we also noted that aberrant splicing was inhibited in PD-treated MDA-MB-231 cells through decreasing spliceosome-involved proteins (Fig. 4d). Specifically, among the 45 downregulated DEPs (Table S5) enriched in spliceosome, 39 were upregulated in TNBC patients (9 were shown in Fig. 4e and f). The results suggested that PD treatment could reduce aberrant splicing by downregulating spliceosome proteins in MDA-MB-231 cells.

### NOMO2/3 May Be the Targets of PD

**Fig. 5:**
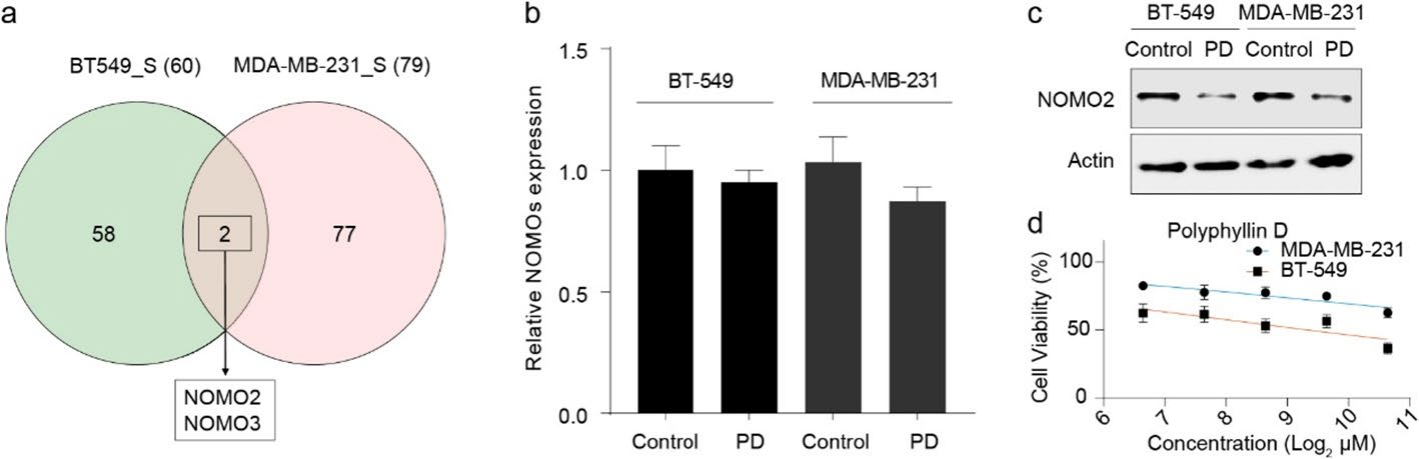
NOMO2/3 may be a target of PD. **a** Quantitative Venn diagram showing the overlapping proteins with significant alterations in BT-549 cells and MDA-MB-231 cells. NOMO2/3 was identified as the potential target of PD. **b** The mRNA level of NOMOs in PD-treated and untreated TNBC cells. There is no significant difference between treatment group and control. **c** The protein level of NOMO2 in PD-treated and untreated TNBC cells. PD could decrease the protein level of NOMO2. **d** The resulting IC_50_ values upon PD treatment of the individual NOMO2-overexpressed cell lines after 24 h. BT-549 and MDA-MB-231 cells were treated with indicated concentrations of PD. PD concentrations in **b** and **c** are IC_50_ for BT-549 and MDA-MB-231, respectively, and treatment duration is 24 h. All data were measured in three independent experiments

To identify the proteins regulated by PD in both of BT-549 cells and MDA-MB-231 cells, we compared the DEPs between PD-treated and untreated BT-549 cells and MDA-MB-231 cells. We found nodal modulator (NOMO) 2 and 3 were all significantly downregulated in two cells after PD treatment (Fig. 5a). NOMO is one of type I transmembrane (TM) proteins [24], which are three isoforms with highly homogenous structures, namely NOMO1/2/3. These three proteins are identical in length, share 99.5–99.8% similar sequences, and have been demonstrated to mediate a wide range of biological processes such as tumor formation [25, 26]. To determine how PD downregulated the expression of NOMO2/3, we examined the mRNA and protein level of NOMO2/3 in PD-treated TNBC cells (IC_50_ and 24 h). We found that PD didn’t affect the expression of NOMO2/3 at transcription level (Fig. 5b), while the protein expression of NOMO2 was indeed downregulated, both in BT-549 cells and in MDA-MB-231 cells (Fig. 5c), suggesting that PD may decrease NOMO2 protein level via post-translational modification (PTM). Finally, we examined the anticancer effect of PD on NOMO2-overexpressed MDA-MB-231 cells. We found the IC_50_ of PD on NOMO2-overexpressed MDA-MB-231 cells was significantly increased (Fig. 5d, compared to Fig. 1b). These findings indicated that PD induced TNBC cell apoptosis by targeting NOMO2/3.

## Discussion

Multiple previous studies demonstrated the anti-proliferation and pro-apoptotic effect on cancers (including breast cancer) of Polyphyllin D [7]. However, the mechanism is unclear. Siu et al. ever-studied MS/MS data of human non-small cell lung cancer (NSCLC) cell line (NCI-H460) using MALDI-Q-TOF and reported that PD played the cytotoxic effect through ER-mitochondrial apoptotic pathway [16]. However, the low coverage of their MS data is insufficient to reveal the full scope of PD effects. Here, we performed a deep genome-wide coverage of protein expression quantification in PD-treated cells by LC–MS/MS using mass spectrometer TIMS-TOF Pro. The findings showed that PD could induce apoptosis by re-activating oxidative phosphorylation pathway in BT-549 cells, whereas inhibiting aberrant splicing in MDA-MB-231 cells, indicating the anticancer effects of PD may be in a cell type-specific manner. In addition, we also found the alternative expression of necroptosis-related proteins (such as RIPK1 and MLKL), and pyroptosis-related proteins (such as GSDMD, IL1β, IL18) in our proteomics data, suggesting the anticancer effect of PD on necroptosis and pyroptosis of cancer cells, which need further study.

Cancer cells are capable of survival and rapid proliferation in the tumor microenvironment (TME) through reprogramming their metabolism. For example, they commonly prefer to utilize glycolysis instead of oxidative phosphorylation (OXPHOS), which is known as aerobic glycolysis or Warburg effect [27, 28]. Although, aerobic glycolysis generates less ATP than OXPHOS doses, it reduces ROS level and ROS-mediated DNA damage in cancer cells [29]. Hence, cancer cells tend to utilize glycolysis to increase DNA stability by downregulating OXPHOS. Conversely, if cancer cells are passive to upregulate OXPHOS, the survival and proliferation of cancer cells will be reduced [30]. Some drugs (such as glycolytic agents) have been demonstrated to shift the metabolism of tumor cells from aerobic glycolysis to oxidative phosphorylation as in normal cells. In this process, cancer growth is inhibited and apoptosis is induced [31]. In our study, we found that PD re-activated oxidative phosphorylation pathway by upregulating the expression of related proteins, which were previously downregulated in TNBC patients.

Constitutive RNA splicing is a crucial step in the cascade of RNA processing events [32], which is catalyzed by spliceosomes [33]. It is widely accepted that cancer cells change the expression pattern of proteins in spliceosomes to regulate tumor-related genes expression, leading to the survival and proliferation of cancer cells [34–36]. Consistently, TNBC alter its certain spliceosomal components and splicing factors to meet the need of development and progression, such as enhanced proliferation, inhibited apoptosis, invasion and metastasis, and angiogenesis [32, 37]. For example, apoptosis-related gene Bcl-x contains two isoforms Bcl-xL and Bcl-xS. Bcl-xL plays an anti-apoptosis role, whereas Bcl-xS exerts a pro-apoptotic effect [38, 39]. Several splicing factors (such as SRSF2, HNRNPA1L2, which are identified in our study) are involved to regulate the ratio of Bcl-xL/Bcl-xS. When the ratio of Bcl-xL/Bcl-xS is decreased, tumor cells are prone to apoptosis [40]. In our study, we found that PD inhibit aberrant splicing by downregulating the expression of spliceosome-involved proteins, which were previously upregulated in TNBC patients.

Finally, we attempted to identify the core proteins regulated by PD in both BT-549 and MDA-MB-231 cells. Exhilaratingly, we found that NOMO2/3 were downregulated in both BT-549 and MDA-MB-231 cells treated by PD. Nodal modulator (NOMO) is one of type I transmembrane proteins that mediates a wide range of biological processes such as tumor formation [26]. In the data from TCGA, NOMOs was upregulated in BC, while PD treatment could downregulate the expression of NOMO in BT-549 and MDA-MB-231 cells. We found that MDA-MB-231 is more sensitive to PD than BT-459. The reason we suspected is that the protein expressions of Nodal [41] and NOMO2/3 (Table S1) were significantly higher in BT-549 than those in MDA-MB-231. Previous studies showed that NOMOs could modulate Nodal/TGFβ signaling pathway to regulate the expression of several critical transcription factors, including Mix-like, GATA, Sox, and Fox [26], which were involved in oxidative phosphorylation pathway or spliceosome [42, 43] . Whether NOMOs and related pathways were the upstream of oxidative phosphorylation pathway and spliceosome need further investigation.

## Conclusion

To summarize, a comprehensive proteomics study was performed on PD-treated or untreated TNBC cells. It is evident that the present experimental data explore the anticancer impact of PD extracted from *Paris polyphylla*. The anticancer mechanism of PD pertains to its capability to inhibit survival and proliferation by re-activating oxidative phosphorylation pathway in BT-549 cells and inhibiting spliceosome in MDA-MB-231 cells. These results suggest that the anticancer effect of PD may be in a cell type-specific manner. Additionally, we also find that PD targets NOMO2/3 probably via post-translational modification (PTM). Our findings extended the understanding of PD against TNBC and highlighted the potential targets, NOMO2/3.

### Supplementary Information

Below is the link to the electronic supplementary material.

Supplementary file1 (DOCX 18 KB)

Supplementary file2 (XLSX 1001 KB)

Supplementary file3 (XLSX 27 KB)

Supplementary file4 (XLSX 34 KB)

Supplementary file5 (XLSX 22 KB)

Supplementary file6 (XLSX 50 KB)

## Data Availability

The mass spectrometry proteomics data have been deposited to the ProteomeXchange Consortium (http://proteomecentral.proteomexchange.org) via the iProX partner repository with the dataset identifier PXD040990 (http://proteomecentral.proteomexchange.org/cgi/GetDataset?ID=PXD040990 or https://www.iprox.cn/page/project.html?id=IPX0006122000).
