# Supplementary material for: Proteomics Analysis of Polyphyllin D-Treated Triple-Negative Breast Cancer Cells Reveal the Anticancer Mechanisms of Polyphyllin D
Source: Appl Biochem Biotechnol. 2023 Aug 25;196(6):3148–61. doi: 10.1007/s12010-023-04679-4 (PMC11166742; doi:10.1007/s12010-023-04679-4)
Supplement: Supplementary file 1 — Supplementary file1 (DOCX 18 KB) [file 12010_2023_4679_MOESM1_ESM.docx]

Proteomics analysis of Polyphyllin D-treated triple-negative breast cancer cells reveal the anticancer mechanisms of Polyphyllin D

Chuanchao Wei^1,#^, Anwei Mao^1,#^,Yongzhi Liu^2,#^, Qing Zhang^1^, Gaofeng Pan^1^, Weiyan Liu^1^, Jiazhe Liu^1,*^

^1^ Institute of Fudan-Minhang Academic Health System, Minhang Hospital, Fudan University, Shanghai, China

^2^ Department of General Surgery, Affiliated Xiaoshan Hospital, Hangzhou Normal University, Zhejiang, China

^#^ These authors contributed equally

^*^ Corresponding author: Jiazhe Liu, Institute of Fudan-Minhang Academic Health System, Minhang Hospital, Fudan University, Shanghai 201100, China. Email: liujiazhe@fudan.edu.cn

Authors’ Email:

Chuanchao Wei: xuan123sen@sina.com

Anwei Mao: Anwei_mao@fudan.edu.cn

Yongzhi Liu: yongzhiliu2020@126.com

Qing Zhang: 169954824@qq.com

Gaofeng Pan: panda_gaofeng@fudan.edu.cn

Weiyan Liu: weiyanLiu2021@163.com

Jiazhe Liu: liujiazhe@fudan.edu.cn

**Liquid chromatography-tandem mass spectrometry (LC-MS/MS)**

**1. Sample preparation for proteome**

For each group, cells were collected and washed in PBS and subjected to global protein extraction using 8M Urea (PH 8.0) containing protease inhibitor (phenylmethanesulfonyl fluoride, PMSF), followed by 3min of sonication (3s on, 3s off, amplitude 25%). Then the protein concentration was obtained through Bradford quantification assay and 100 μg protein was digested overnight following filter-acid sample preparation (FASP) method with 3.5ug trypsin in 50mM ammonium acid carbonate (PH 8.0) overnight at 37℃. Finally, the purified peptides were acquired after extraction with 50% acetonitrile (ACN) and 0.1% formic acid (FA) following desalination in two layers of Empore 3M C18 disk with 2mg packing (3 μm, 150 Å, Agela) in a pipet tip and dried in a vacuum concentrator (Thermo Scientific).

**2. LC-MS/MS analysis of peptide mixture**

Proteome analysis were processed on a nanoElute-HPLC System (Bruker Daltonics) coupled with a hybrid trapped ion mobility spectrometry quadrupole times-of-flight mass spectrometer (TIMS-TOF Pro Bruker Daltonics, Billerica, MA) via a Captive Spray nano-electrospray ion source. Peptide mixture were re-dissolved in solution A (0.1% FA) and loaded onto the analytical column (75 μm i.d. × 25 cm) and separated with a 60min gradient (2−22% solvent B (ACN with 0.1% formic acid) for 45 min, 22−37% B for 5 min, 37−80% B for 5 min, and then 80% B for 5 min) at a flow rate of 600 nl/min. The MS analysis was performed by scanning 100-1700 m/z in positive electrospray mode. The accumulation and ramp time were set as 100 ms each. Survey full-scan MS spectra (m/z 100–1700) were obtained. The ion mobility was scanned from 0.7 to 1.3 Vs/cm2. The overall acquisition cycle of 1.16s comprised one full TIMS MS scan and 10 parallel accumulation-serial frag-mentation (PASEF) MS/MS scans. During PASEF MSMS scanning, the collision energy was ramped linearly as a function of the mobility from 59 eV at 1/K0 = 1.6 Vs/cm2 to 20 eV at 1/K0 = 0.6 Vs/cm2. The mass spectrometry proteomics data have been deposited to the ProteomeXchange Consortium (http://proteomecentral.proteomexchange.org) via the iProX partner repository with the dataset identifier PXD040990.

**3. Proteome identification and quantification**

MS raw files were searched against the Swiss-Prot database (downloaded on August 20, 2020, containing 20, 375 protein sequence entries) using PEAKS Online Xpro Software (v1.4) for peptide and protein identifications. Trypsin was selected as the proteolytic enzyme, and three missed cleavages sites were allowed. The mass tolerance was 15 ppm for precursor and 0.05 Da for production. The oxidation of Methionine and N-acetylation were set as the variable modifications. The false discovery rates of the peptide-spectrum matches (PSMs) and proteins were set to less than 1%. For the proteome quantification, the area values under the curve (AUC) of a peptide feature were subjected to fraction of total (FOT) calculation. Finally, FOT was multiplied by 10^6^ for easy presentation.

**4. Quality control and assessment of LC-MS/MS data**

For the quality control of protein identification, the target-decoy-based strategy was applied to confirm that the FDR (False Discovery Rate) of both peptide and protein were lower than 1%. The program percolator was used to obtain the probability value (q value), and showed that the FDR (measured by the decoy hits) of every peptide-spectrum match (PSM) was lower than 1%. Then all peptides shorter than seven amino acids were removed. The cut-off ion score for peptide identification was 20. All of the PSMs in all fractions were combined for protein quality control, which was a stringent quality control strategy. The q values of both target and decoy peptide sequences were dynamically increased until the corresponding protein FDR was less than 1% employing the parsimony principle.

Dip statistic test and correlation evaluation implemented in R v.4.0.2 were adopted to evaluate the batch effects and sample repeatability in our study. The density plots of all proteins exhibited an expected unimodal distribution by dip statistic test, indicating that the samples passed the quality control. The Spearman’s correlation coefficient was computed for all samples. The average correlation coefficient among the samples was 0.90, indicating the high repeatability of the data.
